# Graphitic and oxidised high pressure high temperature (HPHT) nanodiamonds induce differential biological responses in breast cancer cell lines†
†Electronic supplementary information (ESI) available: Supplementary methods and figures. See DOI: 10.1039/C8NR02177E


**DOI:** 10.1039/c8nr02177e

**Published:** 2018-06-19

**Authors:** Benjamin Woodhams, Laura Ansel-Bollepalli, Jakub Surmacki, Helena Knowles, Laura Maggini, Michael de Volder, Mete Atatüre, Sarah Bohndiek

**Affiliations:** a Cavendish Laboratory , Department of Physics , University of Cambridge , JJ Thomson Avenue , Cambridge , CB3 0HE , UK . Email: seb53@cam.ac.uk ; Email: ma424@cam.ac.uk; b Cancer Research UK Cambridge Institute , University of Cambridge , Li Ka Shing Centre , Robinson Way , Cambridge , CB2 0RE , UK; c Institute for Manufacturing , Department of Engineering , University of Cambridge , 17 Charles Babbage Rd , Cambridge , CB3 0FS , UK

## Abstract

Nanodiamonds have demonstrated potential as powerful sensors in biomedicine, however, their translation into routine use requires a comprehensive understanding of their effect on the biological system being interrogated. Under normal fabrication processes, nanodiamonds are produced with a graphitic carbon shell, but are often oxidized in order to modify their surface chemistry for targeting to specific cellular compartments. Here, we assessed the biological impact of this purification process, considering cellular proliferation, uptake, and oxidative stress for graphitic and oxidized nanodiamond surfaces. We show for the first time that oxidized nanodiamonds possess improved biocompatibility compared to graphitic nanodiamonds in breast cancer cell lines, with graphitic nanodiamonds inducing higher levels of oxidative stress despite lower uptake.

## Abstract 2

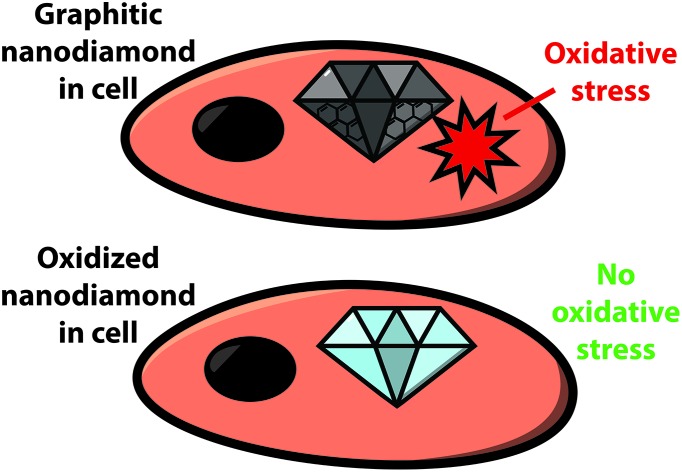
We show here that oxidised nanodiamonds show improved biocompatibility compared to graphitic nanodiamonds when applied in living cells.

## Introduction

Nanodiamonds have been proposed as a reliable and capable replacement for fluorescent dyes in biomedical applications. Nanodiamonds can contain substitutional atoms and vacant lattice sites that result in photostable fluorescent color centers. For example, the nitrogen-vacancy (NV) centers^1^ have been used with cells *in vitro* for tracking,^2^ temperature sensing,^3^ and magnetic field measurement.^4^,^5^ NV color centers also are able to measure electric fields,^6^,^7^ pressure,^8^ pH 9 and nuclear magnetic resonance spectra.^10^,^11^ Owing to their superior spin properties over detonation nanodiamonds, High-Pressure High-Temperature (HPHT) nanodiamonds are commonly exploited for these measurements.^12^,^13^

After HPHT and detonation nanodiamond fabrication, the nanodiamond surface is typically a layer of sp^2^ graphitic carbon.^14^–^16^ For metrology in cells, this graphitic layer is often removed by oxidation, which has been to shown to: reduce charge switching between the NV^–^ and NV^0^ charge states;^17^ improve brightness;^18^ and facilitate surface functionalization to target nanodiamonds to particular intracellular sites such as organelles.^19^,^20^ Identifying and understanding any cellular perturbations caused by the biological application of nanodiamonds with different surface chemistries is vital.

The ability to perform intracellular measurements using nanodiamonds relies firstly on a robust knowledge of the processes that govern their internalization and retention. Both graphitic and oxidized nanodiamonds have been observed to be internalized,^21^,^22^ with oxidized nanodiamonds explicitly shown to be actively internalized by clathrin-mediated endocytosis.^23^ Oxidized nanodiamonds also appear to enhance uptake of various pharmaceuticals and their corresponding efficacy.^24^ The rate at which graphitic and oxidized nanodiamonds are expelled from cells has been reported to be slow, with only about 15% oxidized nanodiamonds expelled after six days in HeLa cells.^21^,^25^

Next, consideration must be made of their potential cytotoxicity. Both graphitic and oxidized nanodiamonds have been demonstrated to have little or no short-term cytotoxicity in human cells in complete culture media,^26^–^32^ although there have been cytotoxic effects observed in bacteria with both surface types.^33^ Many studies have focused on short-term viability; for the longer term experiments enabled by the chemical- and photo-stability of nanodiamonds, a greater impact may be observed on proliferation over time, where slow cell division and death processes can be examined. Application of graphitic nanodiamonds in serum-free media over 24, 48 and 72 h has been shown to reduce cell number,^34^ although a similar study at 24 h for graphitic and oxidized nanodiamonds did not observe a significant effect.^31^ Furthermore, in full medium over 48 h, oxidized diamonds have been shown to have little influence on cell number.^35^ In addition to changes in cellular proliferation, nanoparticles may cause transient stress responses,^36^,^37^ which have yet to be fully explored for nanodiamonds. For example, oxidative stress, an imbalance of free radical species and antioxidants, is an important parameter that is linked to many cell processes such as apoptosis, DNA degradation, as well as cardiovascular and neurodegenerative diseases, and cancer.^38^,^39^ If nanodiamonds are to be exploited as a potential replacement for fluorescent dyes, they should not only be benign in terms of their impact on proliferation, but they should also avoid induction of cellular stress responses. There have been a limited number of studies of nanodiamond induced oxidative stress responses; while unmodified detonation nanodiamonds showed a small antioxidant effect,^40^ oxidized detonation diamonds were found to cause a low level of reactive oxygen species generation in one cell line.^32^ Detonation nanodiamond is often compositionally more impure than HPHT nanodiamond, likely changing how the biological impact.^41^ Acid-oxidized diamonds were observed to have no effect on unstressed neural cells and actually reduced the stress in stressed cells.^42^

Here, we sought to determine the biological impacts of both graphitic and oxidized HPHT nanodiamonds by analyzing cellular uptake as well as proliferative and stress responses in two breast cancer cell lines. We focus on HPHT nanodiamond rather than detonation diamond due to the aforementioned advantageous sensing capability. We present the first direct comparison of graphitic and oxidized HPHT nanodiamonds using realistic concentrations for single cell measurements (≤1 μg mL^–1^), rather than the higher concentrations typically used for drug delivery.

## Materials and methods

### Nanodiamond preparation

Nanodiamonds (Non-detonation, NaBond Technologies Co., China) were manufactured by a High-Pressure High-Temperature (HPHT) bulk diamond process then milled to a nominal 45 nm, with less than 50 ppm nitrogen impurities and containing fluorescent nitrogen-vacancy centers. Oxidation to remove the graphite by burning was performed in a Vecstar VTF1SP tube furnace in air, calibrated with a K-type thermometer to 445 ± 5 °C. Nanodiamonds were drop-cast onto a quartz coverslip (CFQ-2520, UQG Optics, UK) or contained in a crucible (SS22, Almath, UK). The sample was heated in air at 450 °C for 5 hours to remove the graphitic shell (ESI Fig. 1†).

### Nanodiamond characterization

Thermogravimetric Analysis (TGA) was performed with 14 mg of nanodiamond powder (TGA 4000, PerkinElmer, USA). To evaluate optimal conditions for oxidation, the heating temperature was increased at 3 °C min^–1^ or 1 °C min^–1^ up to 900 °C in air and the weight was measured every second. A plateau was observed at 650 °C. The temperature was held at 120 °C for 30 minutes to remove water. Heating at 3 °C per minute decreased the nanodiamond mass remaining from 75% to 25% between 546 ± 1 °C and 592 ± 1 °C. Upon slower heating at 1° per minute, the nanodiamonds were reduced from 75% to 25% mass between of 534 ± 1 °C to 545 ± 1 °C (ESI Fig. 1a†). The largest mass loss rate occurs at a consistent temperature, 537 ± 1 °C (3 °C min^–1^) and 535 ± 1 °C (1 °C min^–1^), as an exothermic reaction occurs to sustain a burning phase. These results place an upper bound on the temperature required to conserve most of the sample mass in a heating plateau profile at 535 ± 1 °C. Considering the derivative of this curve (ESI Fig. 1b†), 425–450 °C appears to be sufficiently far below the onset of burning to perform the oxidation.

For preparation of oxidized nanodiamonds for the experiments in the remainder of this study, the temperature was increased to 450 °C in 5 min, and then held at that level while the mass change was monitored at 1 s intervals (ESI Fig. 1c†). The instrument temperature was calibrated by alumel, perkalloy and iron magnetic phase transitions at the temperatures 154.2 °C, 596 °C and 780 °C to be accurate to ±1 °C across this range. The error in the mass fraction decrease is given by combining the errors in the linear fit to the nominal error in mass accuracy of 0.02%.

To assess the impact of the oxidation process on the nanodiamond chemistry, optical spectroscopy was performed. Raman micro-spectroscopy was performed with a WITec Alpha 300 R Confocal Raman microscope (WITec GmbH, Germany). Samples were prepared by drop-casting diamonds onto a calcium fluoride substrate (CAFP25-1U, UQG Optics, UK) and Raman spectra were recorded using 4 mW 488 nm excitation (CMX1-04813, Newport Corporation, USA) and a 20 × NA = 0.4 objective (421350-9970-000, Nikon, Japan). Data were collected across a 400 μm × 400 μm area in 1 μm steps with 0.5 s integration time per point and calibrated by measuring a silicon standard.

Fourier-Transform Infrared (FTIR) spectroscopy was conducted with a Nicolet iS10 transmission instrument (ThermoFisher Scientific, USA) with the sample of nanodiamonds on NaCl windows (Z527130, Sigma-Aldrich, USA). 64 scans per sample were taken with 2 cm^–1^ resolution. For each measurement, a background of air and an empty slide was used. FTIR spectra were processed by subtracting a cubic polynomial background in the ranges 700–3100 cm^–1^ and 3800–4000 cm^–1^, and smoothed using a Savitsky-Golay filter (width 201, order 2).

To assess the impact of the oxidation process on the nanodiamond sizes, Atomic Force Microscopy (AFM) and Dynamic Light Scattering (DLS) were performed. Atomic Force Microscopy (AFM) was performed with an Oxford Instruments Asylum MFP-3D AFM System. Firstly, two intersecting scratches were made with a diamond cutter onto a quartz substrate (CFQ-1250, UQG optics, UK) for localization. The substrate was cleaned in acetone then isopropanol in a Sonorex Digital 10P Ultrasonic bath at 63 °C (BandelinGmbH, Germany) for 30 minutes each. The nanodiamonds were suspended in ethanol at 1.6 mg mL^–1^ and sonicated for 90 minutes at 320 W of ultrasonic power before deposition on the quartz by a respiratory nebulizer (U22, Omron Healthcare Co., Kyoto, Japan). A 15 μm × 15 μm, 4096 × 4096 scan was taken at 0.2 Hz per line with Nanoworld Arrow-FM-20 Force Modulation tips using non-contact air topographical imaging.

AFM data were processed in Gwyddion (GNU General Public License at http://gwyddion.net/). At least 861 nanodiamonds were assessed. The data were linearly subtracted during collection to correct for tilt. Each image was rotated by 2.2° for mutual alignment and an identical background area on each was set to zero. The images were cropped to show the same diamonds. Data were collected by applying a threshold mask that selected all observed diamonds (3 nm for each), then all diamonds in the shadow of the large particles were removed and the maximum height of the remaining particles was exported. The error in the mean was calculated by dividing the standard deviation by the root of the population size and the distributions were compared by the Mann–Whitney U test.

Dynamic Light Scattering (DLS) data were collected with a Malvern Zetasizer Nano ZSP instrument. Samples were vortexed for 2 minutes at 2025 Hz on a VWR Mixer (444-0203, VWR International Ltd, USA), followed by sonication in an Ultrawave U300H (Ultrawave Ltd, UK), a repetition of vortexing, then filtration through a 0.45 μm Polyethersulfone syringe filter (514-0075, VWR International Ltd, USA). The sample was placed within a plastic cuvette for the experiment (67.754 Polystyrene, Sarstedt AG & Co., Germany) with 637 nm excitation and collection *via* 173° backscattering.

### Cell culture

MDA-MB-231 and MCF-7 adherent breast cancer cells were grown in phenol-free Dulbecco's Modified Eagle Medium (DMEM, 11880-028, ThermoFisher Scientific, USA) with 10% heat inactivated Fetal Calf Serum (FCS, 1050064, ThermoFisher Scientific, USA). The cells were split at 80% confluence in ratios 1 : 20 for MDA-MB-231s and 1 : 10 for MCF-7s. MDA-MB-231 and MCF-7 origins were verified by short tandem repeat genotyping (performed at the Cancer Research UK Cambridge Institute, UK).

### Calculation of number of nanodiamonds per cell

The number of nanodiamonds per cell was calculated using the following parameters. *C*: concentration = (0.01, 0.1 and 1 μg mL^–1^); *V*: volume of medium in a well = 0.3 mL; *L*: mean characteristic dimension of the individual nanodiamonds^43^ = 23 nm; *ρ*: density of diamond = 3.5 × 10^3^ kg m^–3^; and *N*: number of cells per well = 3 × 10^5^.
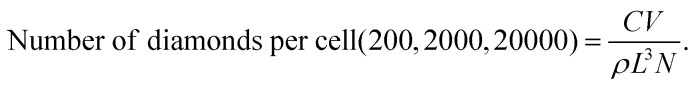


### Uptake experiments

Cells were seeded into six optical-quality eight-well plates (IB-80826, ThermoFisher Scientific, USA) at 5 × 10^4^ cells per well, and incubated overnight to allow for attachment. Graphitic and oxidized nanodiamonds at 1 μg mL^–1^ in 300 μL medium with serum were added to three wells each per plate, and fresh medium without nanodiamonds was added to two wells per plate as controls. The plates were left for 1, 2, 4, 8, 24 and 48 hours. At the completion of the uptake time, the wells were washed 3× in medium. They were then fixed in paraformaldehyde (HT5011, Sigma-Aldrich, USA) for 15 minutes, followed by three washes in Hank's Balanced Salt Solution (14025092, ThermoFisher Scientific, USA). Cell nuclei and membranes were stained by addition of 3 mL HBSS containing 2 drops of NucBlue Live Ready Probes Reagent (R37605, Invitrogen, USA) and 5 μg mL^–1^ Wheat Germ Agglutinin – AlexaFluor 488 membrane dye (W11261, Invitrogen, USA) simultaneously for 10 minutes. The wells were then washed in phosphate buffered saline (PBS) (2×) and suspended in PBS. The fixed cell samples were scanned with an Olympus Confocal Fluorescence Microscope FV1200, using a 60× oil objective, a pixel size of 132 nm, a scan speed of 4 μm s^–1^ and a confocal size of 71 μm with a resolution of better than 215 nm. Bright field illumination was used to locate cells, so that at least 175 nuclei were captured per condition. The *z* position was set by moving upwards until the substrate was not observed. The samples were sequentially raster scanned with 405 nm, 488 nm and 633 nm lasers to the collect the nuclear, membrane and nanodiamond signal respectively. Nanodiamond aggregates were detected by elastic light scattering around 633 nm using a collection filter 575–675 nm.

### Proliferation

Cellular proliferation measurements were conducted with an Incucyte Zoom System (Essen Bioscience, USA). Four replicates of 0, 0.01, 0.1 and 1 μg mL^–1^ nanodiamonds were added to MCF-7 or MDA-MB-231 cells in phenol-free DMEM media supplemented with Glutamax (35050-038, ThermoFisher Scientific, USA) and 10% FCS. A positive control for proliferation change was induced using 1 mM H_2_O_2_ (386790-100, VWR, UK). Images were analyzed with the Incucyte Zoom software version 2016, and a size threshold of 350 μm^2^ was used so that nanodiamonds were not counted as cells.

### Oxidative stress

Cells were seeded into eight-well plates at a density of 5 × 10^4^ cells per well. Cells were incubated overnight and then washed in DMEM/F-12 medium (21041-025, ThermoFisher Scientific, USA) with serum. Graphitic and oxidized diamonds were each added into two wells at 1 μg mL^–1^ in 300 μL media with serum and incubated for four hours. As a positive control to induce oxidative stress, 200 μM *tert*-Butyl hydroperoxide (TBHP) was added to two wells one hour before imaging. Just prior to imaging, media was removed from all wells and was replaced by media containing two drops of NucBlue dye according to the recommended protocol and 0.5 μM CellRox™ Orange Reagent (C10443, Invitrogen, USA) for 30 minutes. Cells were then washed in fresh media and imaged live at 37 °C and 5% CO_2_ using an excitation laser at 405 nm with collection within 425–475 nm, and an excitation laser at 559 nm with detection within 570–670 nm. A scan speed of 10 ms per pixel at a confocal aperture of 105 μm were used to capture images (512 × 512 pixels).

### Data and image analysis

Raman spectra were analyzed using *k*-means clustering (WITec Project 4, Witec GmbH, Germany) to extract the diamond signal. Fluorescence background was removed by fitting and subtraction of a 5th order polynomial between 800–1300 cm^–1^ and 1710–2500 cm^–1^. Data were normalized to the integral of the pure diamond peak between 1280–1390 cm^–1^. The relative amount of graphite on the sample was calculated by integration between 1490–1660 cm^–1^ and this wavenumber range was used for *p*-value significance, error calculation and percentage reduction. The area was calculated by using trapezium rule, and its error was estimated by the standard deviation of the data around a cubic fit. *P*-Value significance was investigated by a two-tailed Mann–Whitney U test as the data did not pass a normality test.

Proliferation tests were analyzed with standard ANOVA. Where data sets violated the assumptions of a standard ANOVA under the homogeneity of variances condition, in the case of CellRox data, Kruskal–Wallis ANOVA and Dunnett's *post-hoc* test were used instead. Microscopy images were processed using custom software written in FIJI ImageJ.^44^ For the uptake experiment, image filenames were initially blinded and artifacts were removed, including the scattering signals from fragmenting nuclei. After this, nuclear areas were measured and counted using the ‘Thresholding’ and ‘Particle Analyzer’ (>50 μm) functions. The membrane stain was used to define the edges of cells, and filled to designate areas enclosed by membrane. These areas were then removed if found not contain a nucleus (Binary Feature Extractor^45^). The nanodiamond scattering channel was thresholded by considering nine representative images and setting the value as closely as possible for both cell lines. All diamonds that were not totally contained within cells were removed (Binary Feature Extractor, 100% overlap^45^) and the remaining particles were analyzed. Two factor ANOVA *via* Regression was applied to account for unbalanced control with *post-hoc t*-tests under Dunn-Šidák were used.

## Results

### Nanodiamond preparation and characterization

**Fig. 1:**
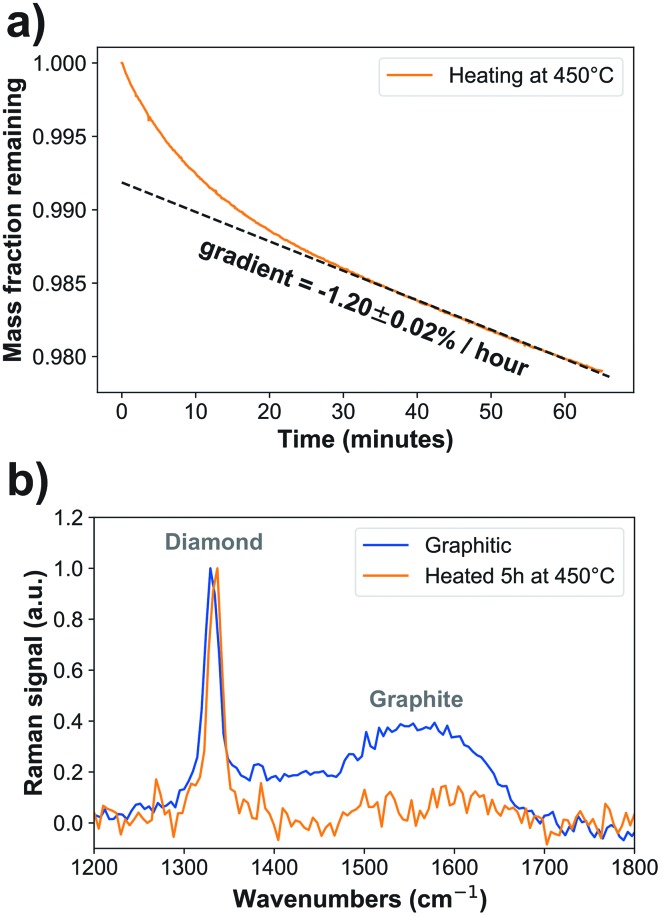
Graphite is effectively removed by heating at 445 ± 5 °C for 5 hours. (a) Thermogravimetric analysis shows that mass is slowly lost at a rate of –1.20(2)% per hour. *N*_Sample_ = 1. (b) Raman spectra show that the graphitic signal (1575 cm^–1^) is reduced relative to the diamond signal (1332 cm^–1^) under heating conditions. At 445 ± 5 °C, the area under the curve is reduced by 76 ± 9% indicating the sample has been oxidized (*p* = 2.2 × 10^–12^). *N*_DataPoints_ = 16 000.

Oxidized nanodiamonds (ESI Fig. 2a†) were prepared by heating in air using conditions optimized *via* thermogravimetric analysis to achieve low mass loss of approximately 2% over one hour, converging to a linear rate of –1.20 ± 0.02% per hour (Fig. 1a, ESI Fig. 2b†). Verification of the oxidation process was made using optical spectroscopy. Raman spectroscopy (Fig. 1b) showed that the peak associated with graphitic carbon (1575 cm^–1^) was reduced in relative area under curve compared to that of diamond (1332 cm^–1^) from 51.8 ± 0.5 to 12.2 ± 1.1 (arbitrary units, *p* = 2.2 × 10^–12^). Fourier Transform Infrared Spectroscopy also provided further evidence of oxidation, through increased carboxyl C=O (1786 cm^–1^) and C–O–C peaks (1089 cm^–1^) relative to C=C between 1430 cm^–1^ and 1490 cm^–1^ (ESI Fig. 2c and d†). Also, as expected, the spectrum exhibited a shift in the C=O groups from aldehyde (1721 cm^–1^) to carboxyl (1784 cm^–1^) as the groups were converted into the most oxidized form through the heating process.^15^,^33^,^46^–^50^

We next analysed the change in the size of the nanodiamonds with oxidation. The mean size determined using AFM before oxidation was 8.1 ± 0.2 nm, and after oxidation it was 7.5 ± 0.2 nm, a reduction of 0.6 ± 0.2 nm (ESI Fig. 3a†). From TGA we expect to see a ∼6% mass decrease after five hours, which would correspond to a ∼0.2 nm radius decrease on a spherical 7.6 nm nanodiamond, which is consistent with these AFM findings. We also examined the aggregation of the nanodiamonds through DLS and confocal optical microscopy. Examination of the size distribution of nanodiamonds suspended in water at 66 μg mL^–1^ found that oxidized nanodiamonds tend to aggregate into significantly smaller clumps than graphitic nanodiamonds (ESI Fig. 3b†). The aggregation of nanodiamonds is further exacerbated by suspension in the complete media used for cell culture studies, where aggregates of up to 6 μm in size could be observed; again, oxidized nanodiamonds showed a smaller aggregate size compared to graphitic nanodiamonds (ESI Fig. 3c and d†).

### Nanodiamond uptake in cells

**Fig. 2:**
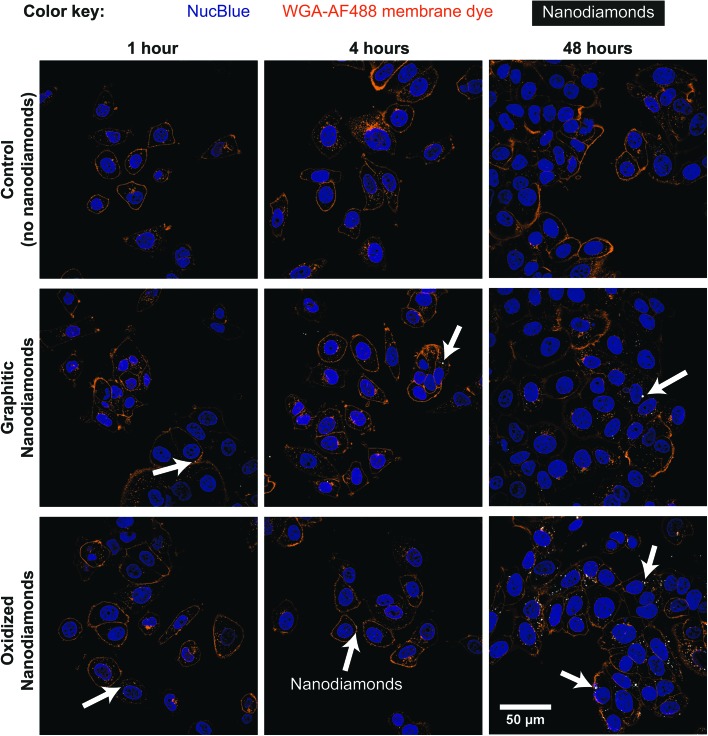
Nanodiamond uptake observed in MCF-7 cells. Cells were incubated with 1 μg mL^–1^ graphitic or oxidized nanodiamonds for 1, 2, 4, 8, 24 and 48 hours before fixation. Cells were then co-stained with NucBlue nuclear stain and membrane stain Wheat Germ Agglutinin-Alexa Fluor 488. Nanodiamond scattering signal was detected at 633 nm. An increase in the number of nanodiamonds within cells may be observed over time up to 48 h (white arrows denote dense cellular uptake).

Cellular experiments were performed using two different breast cancer cell lines. MDA-MB-231 and MCF-7 are breast cancer lines representative for mesenchymal-like and luminal-like cancer types respectively. While MCF-7 cells express estrogen and progesterone receptors and form invasive breast ductal carcinomas, MDA-MB-231 cells do not express estrogen receptors and produce highly invasive and metastatic tumors. Cells were exposed to nanodiamonds for up to 48 h and microscopy images (Fig. 2, ESI Fig. 4†) show a clear increase in the accumulation of nanodiamonds over time, many of which were present as aggregate clumps of similar sizes. A detailed summary of the size distribution of oxidized and graphitic nanodiamond clumps in both cell types can be found in ESI Fig. 5 and 6.† Interestingly, when examining the distributions of particle sizes in cells (ESI Fig. 5 and 6†), the number of particles below 0.02 μm^2^ apparently decreases in MDA-MB-231s over time, whereas the number of particles above 1 μm^2^ increases up to four hours, and then decreases between 8–24 hours. In contrast, MCF-7 cells continuously uptake particles throughout the 48 hours experiment.

**Fig. 3:**
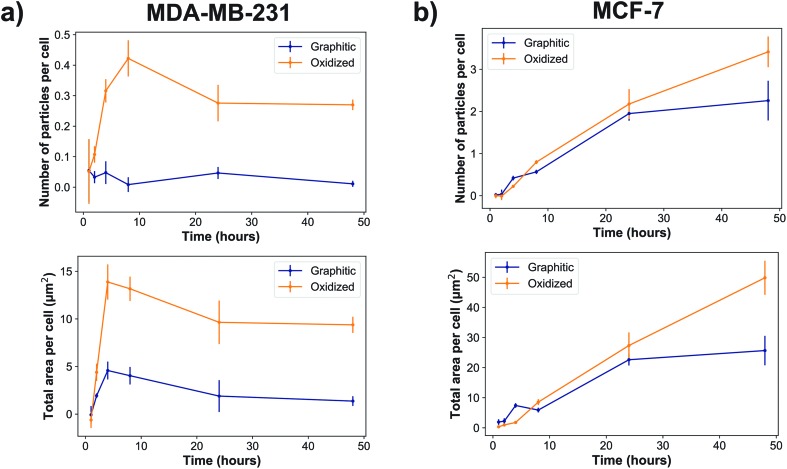
Nanodiamonds were internalized into cells over time in two different breast cancer cell lines: (a) MDA-MB-231 and (b) MCF-7 cells. ‘Total area per cell’ refers to the total nanodiamond intracellular area in the images divided by the number of nuclei observed in that image. Oxidized diamonds were taken up in a greater amount than graphitic diamonds (*p* = 4 × 10^–8^ for MDA-MB-231s and *p* = 3 × 10^–5^ for MCF-7s). *N*_Biologicalreplicates_ = 2 (control) and 3 (nanodiamond sample). *N*_Nuclei_ = 500 ± 200 per condition for MDA-MB-231 and 430 ± 170 per condition for MCF-7.

Quantitatively, our data show that MDA-MB-231 cells showed a significantly higher uptake of oxidized rather than graphitic nanodiamonds (Fig. 3a), with both a greater number of particles (*p* = 4 × 10^–8^), as well as a greater observed area of nanodiamond signal (*p* = 4 × 10^–9^). MDA-MB-231 cell uptake reached a peak after four hours of exposure of 4.7 ± 0.9 μm^2^ per cell and 14 ± 2 μm^2^ per cell average for graphitic and oxidized diamonds respectively. The subsequent decline in signal is likely due to cell division over the following hours. By contrast, MCF-7 cells showed a more similar uptake of graphitic and oxidized nanodiamonds until 24 h, at which point the uptake of graphitic nanodiamonds appeared to reach saturation (Fig. 3b). MCF-7 cells also showed a level of uptake that was an order of magnitude higher than that seen in MDA-MB-231 cells. Nonetheless, oxidized diamond uptake was also significantly higher than graphitic diamond uptake in MCF-7 cells (*p* = 3 × 10^–5^), making this a consistent finding across both cell types.

### Cellular proliferation with nanodiamonds

**Fig. 4:**
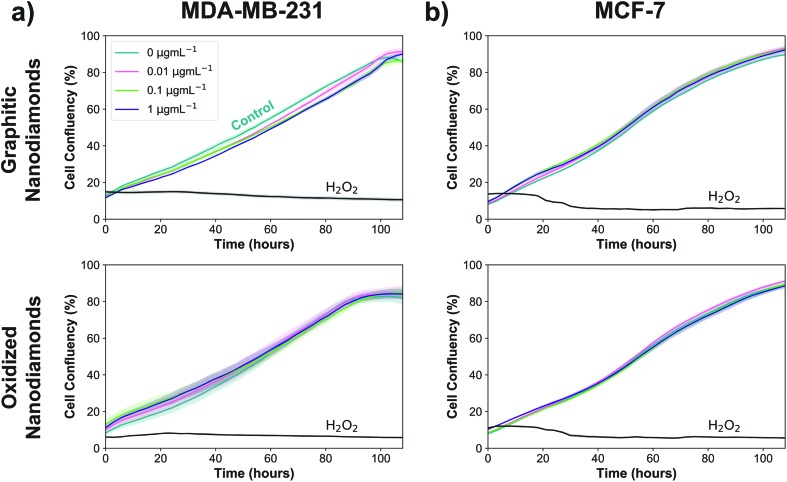
Nanodiamonds have negligible anti-proliferative effect. Graphitic diamonds cause a small decrease in confluency at 1 μg mL^–1^ in MDA-MB-231 cells (top left, –5 ± 2%, *p* = 0.0014), but no other experiments showed any significant decrease at these concentrations. *N*_replicates_ = 4.

Next, experiments were performed to assess whether the uptake of graphitic and oxidized nanodiamonds had any impact on the proliferation of MDA-MB-231 and MCF-7 breast cancer cell lines. We added nanodiamond concentrations of 0, 0.01, 0.1 and 1 μg mL^–1^, corresponding to approximately 200, 2000 and 20 000 nanodiamonds per cell. Hydrogen peroxide was used as a positive control of cell death and phosphate buffered saline as a negative control. We used automated phase contrast microscopy (Incucyte) to count cells and determine confluency. Considering the growth phase between 48 and 108 hours, cell confluency was slightly reduced at 1 μg mL^–1^ for graphitic nanodiamonds on the MDA-MB-231 (Fig. 4a) cells (–5 ± 2%, *p* = 0.0014), but no change was observed with oxidized diamonds (+1 ± 4% *p* = 0.89). MCF-7 (Fig. 4b) cells appeared to be unaffected by the addition of nanodiamonds of either type (graphitic: +2 ± 2% *p* = 0.37, oxidized: –1 ± 3% *p* = 0.73).

### Nanodiamond-induced stress

**Fig. 5:**
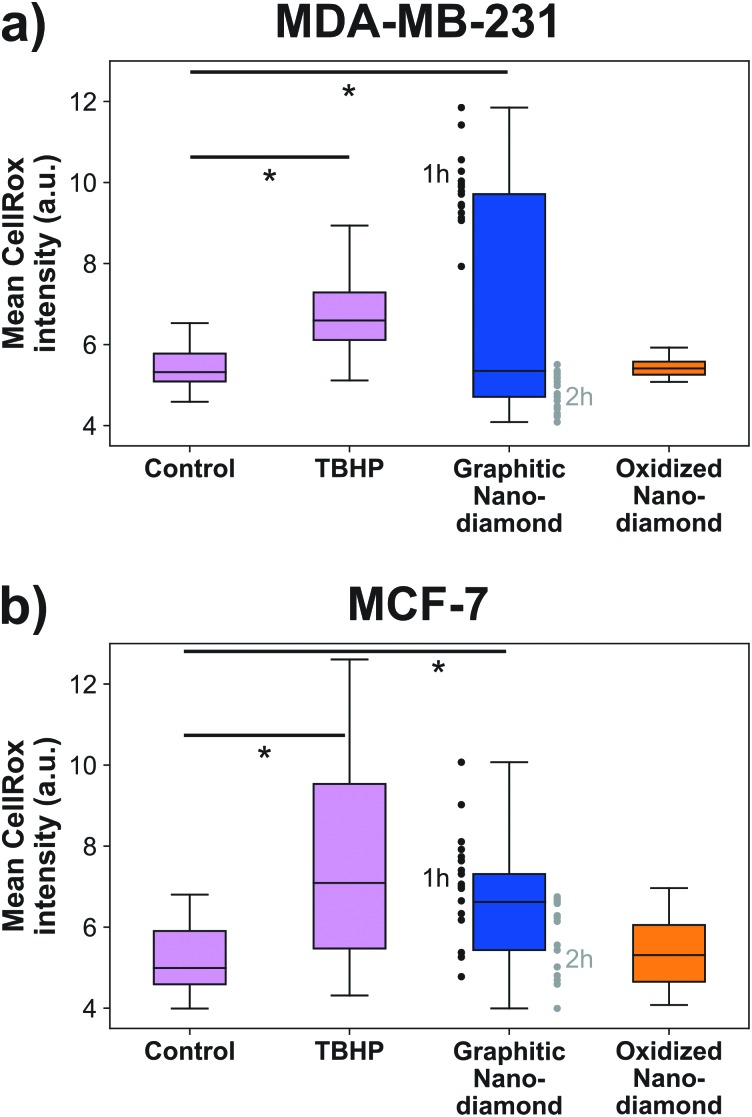
Graphitic nanodiamonds produced increased levels of cellular oxidative stress for (a) MDA-MB-231 cells. Graphitic diamonds had a significant *d*-statistic (= 4.6 > *d*-critical = 2.3) and TBHP performed successfully as the positive control (significant *d*-statistic = 8.7 > *d*-critical = 2.3) (b) MCF-7 cells (significant *d*-statistic = 5.7 > *d*-critical = 2.3). TBHP, the positive control performed as expected 3.6 > *d*-critical = 2.3. Oxidized diamonds did not increase the cellular stress in either cell line. Oxidative stress was generally higher at 1 h time point for graphitic diamonds. Outliers greater than 1.5× the interquartile range were not plotted. *d*-Statistics were used in place of *p* values, as the sample did not reflect a normal distribution, and so the analysis was done on ranked data. *N*_images_ = 24, 31, 33, 15, 25, 24, 33, 31 (column order), *N*_cells_ = 451, 478, 671, 368, 530, 433, 897, 773.

Finally, we examined the cellular oxidative stress responses to evaluate whether any transient effects were present that did not manifest as anti-proliferative. MDA-MB-231 cells (Fig. 5a) showed significant increase in oxidative stress under application of the positive control TBHP and for incubation with graphitic nanodiamonds (ANOVA *p* = 6 × 10^–5^). In *post-hoc* comparison, the critical threshold of the *d*-statistic was 2.3, which was surpassed by the TBHP (*d*-stat = 8.7) and the graphitic diamonds (*d*-stat = 4.6). Oxidized nanodiamonds did not show any effect on oxidative stress, with levels remaining comparable to the control condition (*d*-stat = 1.8). This pattern of response was repeated in MCF-7 cells (Fig. 5b), where TBHP and graphitic diamonds were found to induce stress at a level above the control (*d*-stat = 3.6, *d*-stat = 5.7 respectively) and oxidized diamonds did not show a significant change (*d*-stat = 1.7). Oxidative stress measurements were made at both 1 h and 2 h time points; teasing these apart it was apparent that MDA-MB-231 cells were most stressed in the initial 1 h but had largely returned to normal stress levels after 2 h. MCF-7 cells had a more similar response at both time points. The higher stress levels from graphitic nanodiamonds are of particular note, given the lower uptake of these nanodiamonds into cells.

## Discussion

Nanodiamonds containing NV centers have been explored as a chemical- and photo-stable replacement for fluorescent dyes in cells for applications such as organelle tracking and temperature sensing. Here, we sought to determine the biological impacts of both graphitic and oxidized HPHT nanodiamonds, examining cellular uptake as well as potential impacts on proliferation and importantly, cellular stress responses. We chose to focus on HPHT nanodiamonds as their superior spin properties have led to wide use for biological sensing applications,^5^,^17^,^51^–^53^ both in graphitic^2^ and oxidized forms.^52^ Oxidized forms of nanodiamond have often been used in biological experiments due to their improved functionalization capability, but direct comparison of biocompatibility *via* oxidative stress in graphitic and oxidized HPHT nanodiamonds from the same batch has yet to be reported.

We have shown for the first time that oxidized HPHT nanodiamonds show improved biocompatibility compared to graphitic HPHT nanodiamonds. In breast cancer cell lines, graphitic nanodiamonds induced higher levels of oxidative stress despite lower uptake compared to oxidized nanodiamonds. We observed an order of magnitude greater nanodiamond uptake in MCF-7 cells over time, particularly at the later time points, when compared against MDA-MB-231 cells. This is consistent with the increased expression of clathrin protein in MCF-7 cells,^54^ as nanodiamonds have previously been observed to be mostly internalized by clathrin-mediated endocytosis.^23^ We also observed oxidized nanodiamond being uptaken at a higher rate than graphitic nanodiamonds in both cell lines, but with a more dramatic difference in the MDA-MB-231 cells.

We noted that oxidized nanodiamonds formed smaller aggregates in cell culture media, which may go some way to explain these differences. In particular, at least 72% of oxidized nanodiamond aggregates were below 250 nm in diameter, while at least 56% of graphitic nanodiamond aggregates were below this size. If further size reduction were needed in future applications, it would be possible to use techniques such as bead-assisted sonic disintegration or salt-assisted dry attrition milling for temporary physical separation of the particles,^55^ or the addition of chemicals to the nanodiamond surface such as serum proteins,^56^,^57^ surfactants,^58^ lysine^59^ or various polymers^60^–^64^ for chemical separation.

Uptake dynamics have been shown to be shape dependent in nanodiamonds, with rounder particles remaining in cells for longer,^21^ so it may be that oxidized nanodiamonds form aggregates of different roundness; this would need to be verified in future by a higher resolution technique such as TEM. Furthermore, nanoparticle surface charge can be an important factor in intracellular uptake, although contradictory findings have been reported in the literature with regard to how nanoparticle interactions with the cell surface relates to surface charges.^65^,^66^ Oxidized nanodiamonds have been reported to exhibit more electronegative zeta potentials,^57^,^67^–^69^ so the difference in surface charge between the two nanodiamond types studied here may contribute to the differences in uptake.

In proliferative studies on cells, we observed a small but significant decrease in proliferation under the highest concentration of HPHT graphitic diamonds in MDA-MB-231 cells. No significant difference was observed at any of the tested concentrations of oxidized nanodiamonds. This low cytotoxicity profile is consistent with other published work, which generally shows low or non-existent nanodiamond cytotoxicity,^30^–^32^,^70^–^74^ over similar time courses.^29^,^75^–^77^ Our work on HPHT diamond aligns with studies on detonation diamond despite the compositional differences,^41^ with graphitic nanodiamonds suppressing cell proliferation and oxidized nanodiamonds having the lowest cytotoxic effect.^15^,^28^ Although we did not observe any anti-proliferative effect of oxidized nanodiamonds, we exposed our cells to a concentration range suitable for experiments interrogating local organelle tracking or temperature sensing, rather than those used for drug delivery, which would be substantially higher. At higher concentrations such as 1 μg mL^–1^ effects including apoptosis have been observed even with oxidized nanodiamonds.^78^

Evaluating oxidative stress in cells exposed to nanodiamonds, we observed that graphitic HPHT diamonds cause a high degree of stress in cells, exceeding that of the positive control. This agrees with previous studies where unmodified nanodiamonds were observed to produce oxidative stress,^79^,^80^ though neither the amount of graphite nor the fabrication procedure were defined in these studies. The observation of oxidative stress under graphitic nanodiamonds is also reported for other forms of sp^2^ carbon,^81^–^83^ especially pure carbon black particles.^84^–^86^ Horie *et al.* also examined three varieties of nanodiamonds with different zeta potentials.^75^ Those with positive zeta potentials caused oxidative stress, while the negative ones did not, which may be consistent with our findings. Of particular interest in our results is the fact that we did not observe any oxidative stress when cells were exposed to oxidized diamonds, consistent with previous reports at both the cellular^42^ and organism levels.^87^

Our results therefore suggest that the impact of the surface chemistry of nanodiamonds can be significant for biological applications. It would therefore be prudent for nanodiamonds to be oxidized prior to application in cells, regardless of whether they are being subjected to functionalization. According to our findings, oxidation can increase cellular uptake and minimize any potential oxidative stress response. These benefits exist in addition to the well known benefits of reduced charge switching between the NV^–^ and NV^0^ charge states,^17^ improved brightness^18^ and facilitating surface functionalization for targeting.^19^,^20^

Despite the promising findings of our direct comparison of the biological impacts of graphitic and oxidized nanodiamonds in two different cell types, there remain some limitations to our study. Firstly, biocompatibility is expected to be determined by surface chemistry and shape,^88^ so it is reasonable to expect that differences in the fabrication methods such as HPHT or detonation processes could cause variations in the cellular response. A direct comparison of nanodiamonds of a similar size and shape produced by the two different processes would be of interest in future work. Secondly, to prepare our oxidized nanodiamonds we use oxidation by heating in air alone. While this is a commonly used procedure,^19^ it is also often replaced or combined with cleaning by acids, UV-Ozone, or oxygen plasma, which may exaggerate the differences in surface chemistry, potentially leading to different results in uptake, proliferation and oxidative stress. Thirdly, the optical scattering measurement used in our uptake experiments has been shown to be insensitive below a diameter of 37 nm for nanodiamonds,^89^ so we may be missing signals associated with any free nanodiamonds undergoing passive uptake, as suggested elsewhere.^23^ However, given the size distributions of our nanodiamonds in solution, we expect this to be a relatively minor contribution to the overall signal. Finally, we studied only uptake, proliferation and oxidative stress. Future work should examine other potential sources of stress response, such as inflammatory or genotoxic responses.^79^

## Conclusions

It is vitally important for any sensor used to perform measurements in a biological system to avoid perturbation of the system that it is measuring. We have shown that nanodiamond oxidation, which is used to reduce charge switching between NV^–^ and NV^0^ and to improve brightness, is also advantageous for increasing intracellular uptake and avoiding oxidative stress in cells. Improved biocompatibility is crucial for the use of nanodiamonds as a revolutionary biomedical tool and implies that HPHT graphitic diamonds should first be oxidized for biological metrology, as this greatly reduces the effect that the sensor has on the system it is measuring.

## Author contributions

B. W. and S. B. conceived the experiments, B. W., L. B., J. S., L. M. and H. K. conducted the experiments, B. W. analyzed the results. M. A. and S. B. supervised the project. All authors reviewed the manuscript.

## Conflicts of interest

The authors do not have any conflicts of interest.

## Supplementary Material

Supplementary information
