# Supplementary material for: Graphitic and oxidised high pressure high temperature (HPHT) nanodiamonds induce differential biological responses in breast cancer cell lines
Source: Nanoscale. 2018 Jun 19;10(25):12169–79. doi: 10.1039/c8nr02177e (PMC6034157; doi:10.1039/c8nr02177e)
Supplement: Supplementary file 1 [file NR-010-C8NR02177E-s001.pdf]

Electronic Supplementary Material (ESI) for Nanoscale.
This journal is © The Royal Society of Chemistry 2018

Supplementary Information: Graphitic and oxidised high pressure high temperature (HPHT) nanodiamonds induce differential biological responses in breast cancer cell lines

Benjamin Woodhams[a,b], Laura Ansel-Bollepalli[a,b], Jakub Surmacki[a,b], Helena Knowles[a], Laura Maggini[c], Michael de Volder[c], Mete Atatüre*[a], Sarah Bohndiek*[a,b]

[a] *Cavendish Laboratory, Department of Physics, University of Cambridge, JJ Thomson Avenue, Cambridge, CB3 0HE, UK*
[b] *Cancer Research UK Cambridge Institute, University of Cambridge, Li Ka Shing Centre, Robinson Way, Cambridge, CB2 0RE, UK*
[c] *Institute for Manufacturing, Department of Engineering, University of Cambridge, 17 Charles Babbage Rd, Cambridge, CB3 0FS, UK*

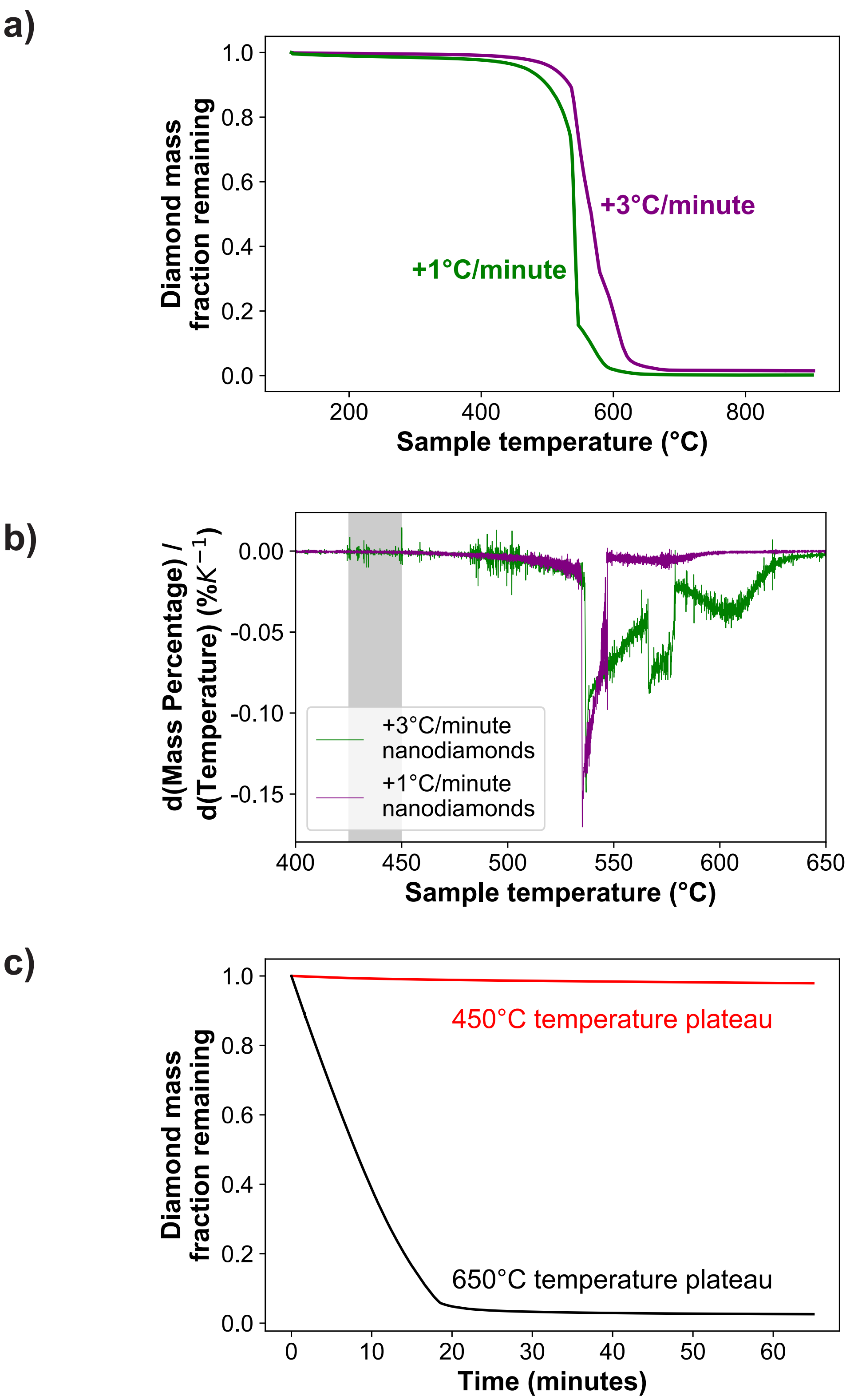

Figure S1: Thermogravimetric analysis to establish appropriate conditions for oxidation. Heating nanodiamonds in air on a weight balance indicates that 450 °C is an appropriate temperature to oxidize the surfaces of nanodiamonds. a) The mass of a nanodiamond sample as the furnace temperature is increased causing the diamonds to burn. b) The mass change plot is the derivative of (a) and shows that the onset of major mass loss occurs at 535 ± 1 °C and 537 ± 1 °C for the two samples. c) Temperature plateaus at 450 °C and 650 °C show that 450 °C only causes slow mass loss. See also main text Figure 1a, where the 450 °C heating is observed to cause mass loss at -1.20±0.02%/hour.

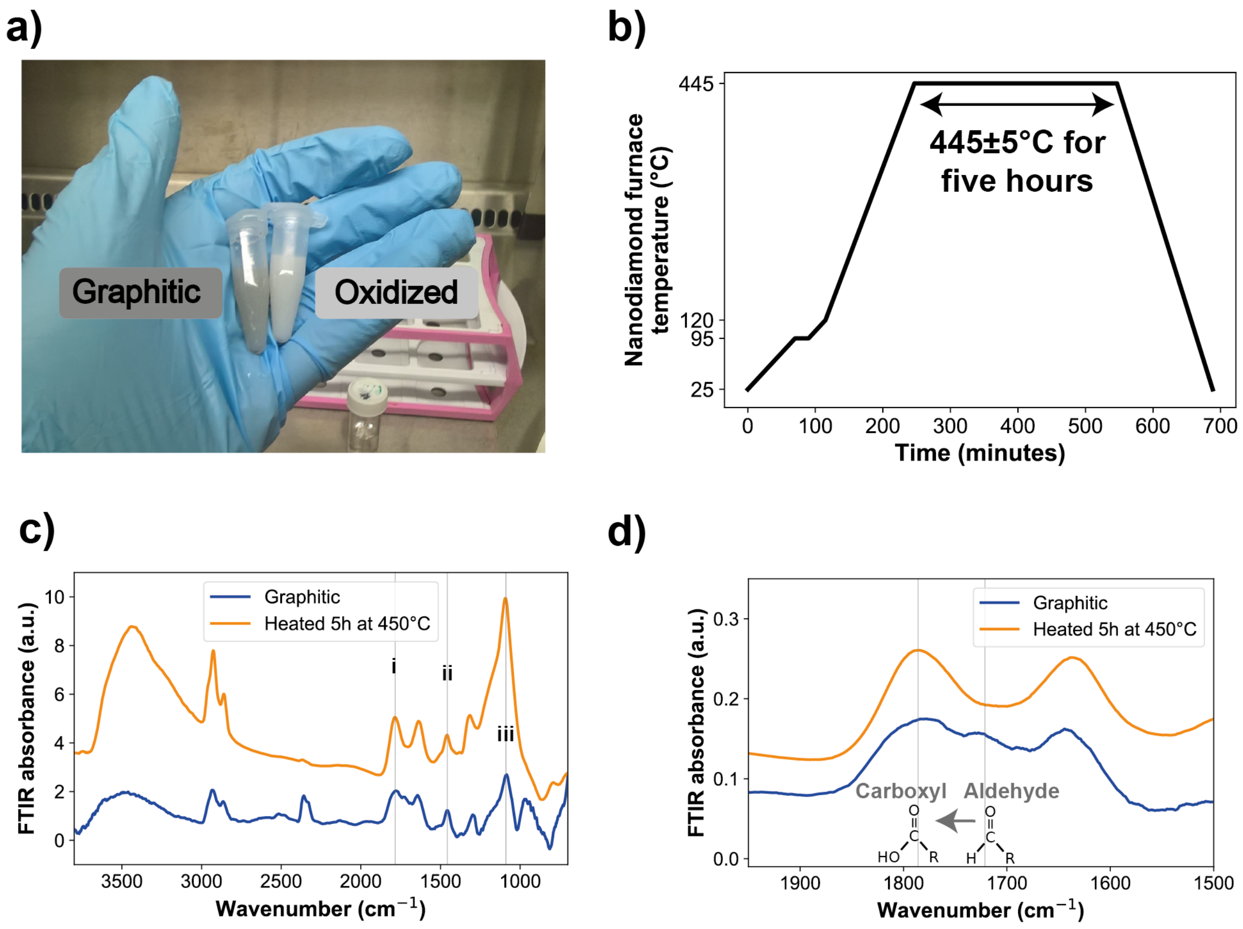

Figure S2: Nanodiamond characterization. a) A photograph of graphitic and oxidized nanodiamonds at 1 mg mL^{-1} in water shows a color change after oxidation. b) Temperature profile for the procedure of nanodiamond oxidation. This was performed in air. c) Fourier Transform Infrared Spectroscopy (FTIR) of nanodiamonds, normalized to the C=C bond peak (ii) between 1430 cm^{-1} and 1490 cm^{-1}. The peaks for carboxyl C=O (i) at 1786 cm^{-1} and C-O-C (iii) 1089 cm^{-1} show a relative increase after oxidation. $N_{\text{Sample}} = 1$. d) A zoomed FTIR spectrum, normalized between between 1550 cm^{-1} and 1900 cm^{-1}. This shows that C=O groups are converted into the most oxidized form through the heating process (1721 cm^{-1} aldehyde to 1784 cm^{-1} carboxyl).

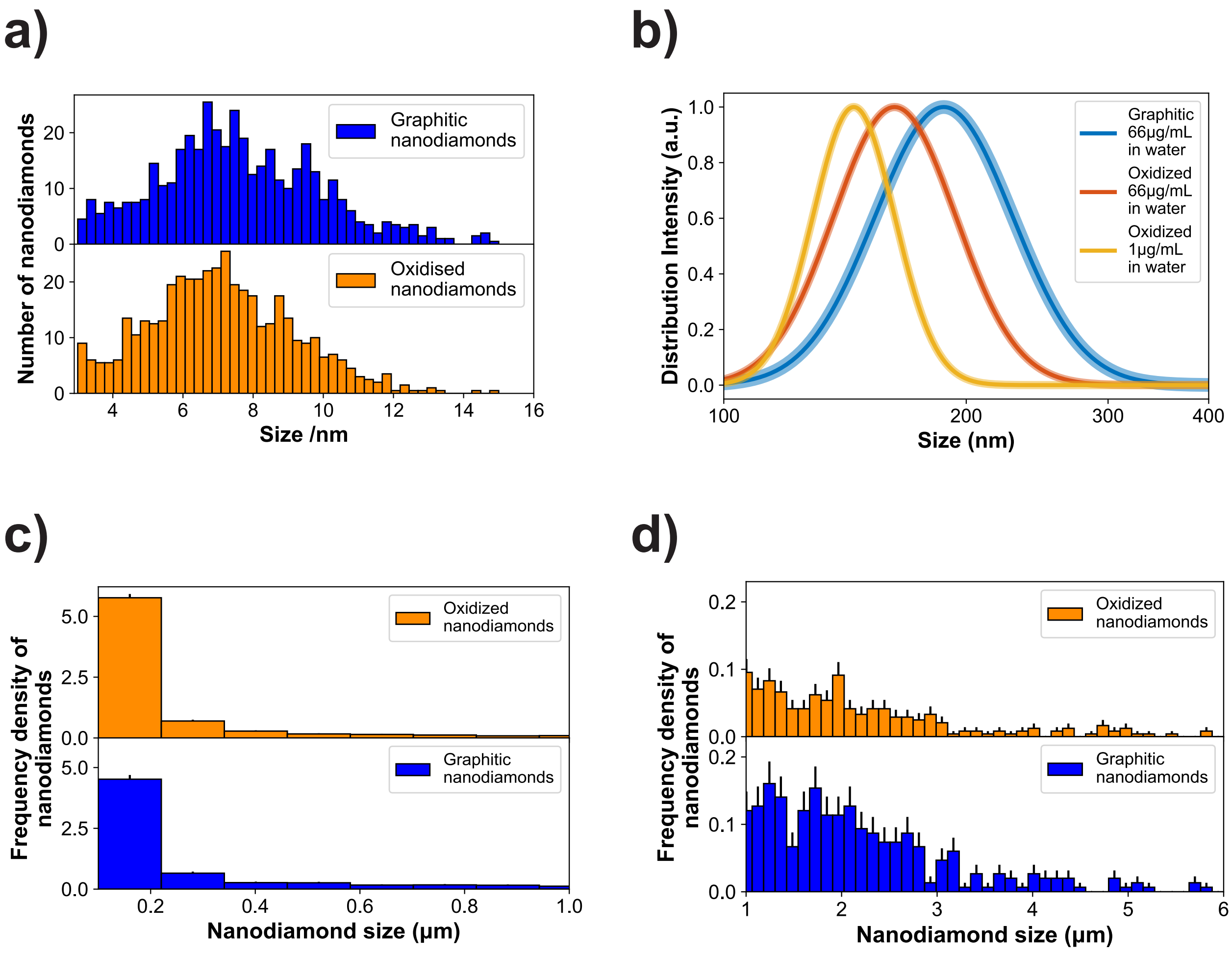

Figure S3: Comparison of nanodiamond size, before and after oxidation. a) Atomic Force Microscopy data from individual nanodiamonds measured in air. b) Dynamic Light Scattering measured in water shows that the aggregate clump size decreases after oxidation and at lower concentration. c) Nanodiamonds were added to DMEM/F-12 medium without cells at a concentration of $1\,\mu g\,mL^{-1}$ and the clumps were observed with confocal optical scattering imaging and plotted between 0.01–1 μm. There was a small reduction in the average size of clumps after oxidation. The percentages of clumps below 250 nm were 56% and 72% for graphitic and oxidized nanodiamonds, respectively. 0.01–1 μm d) The same as c, but expanded to visualize the range from 1–6 μm

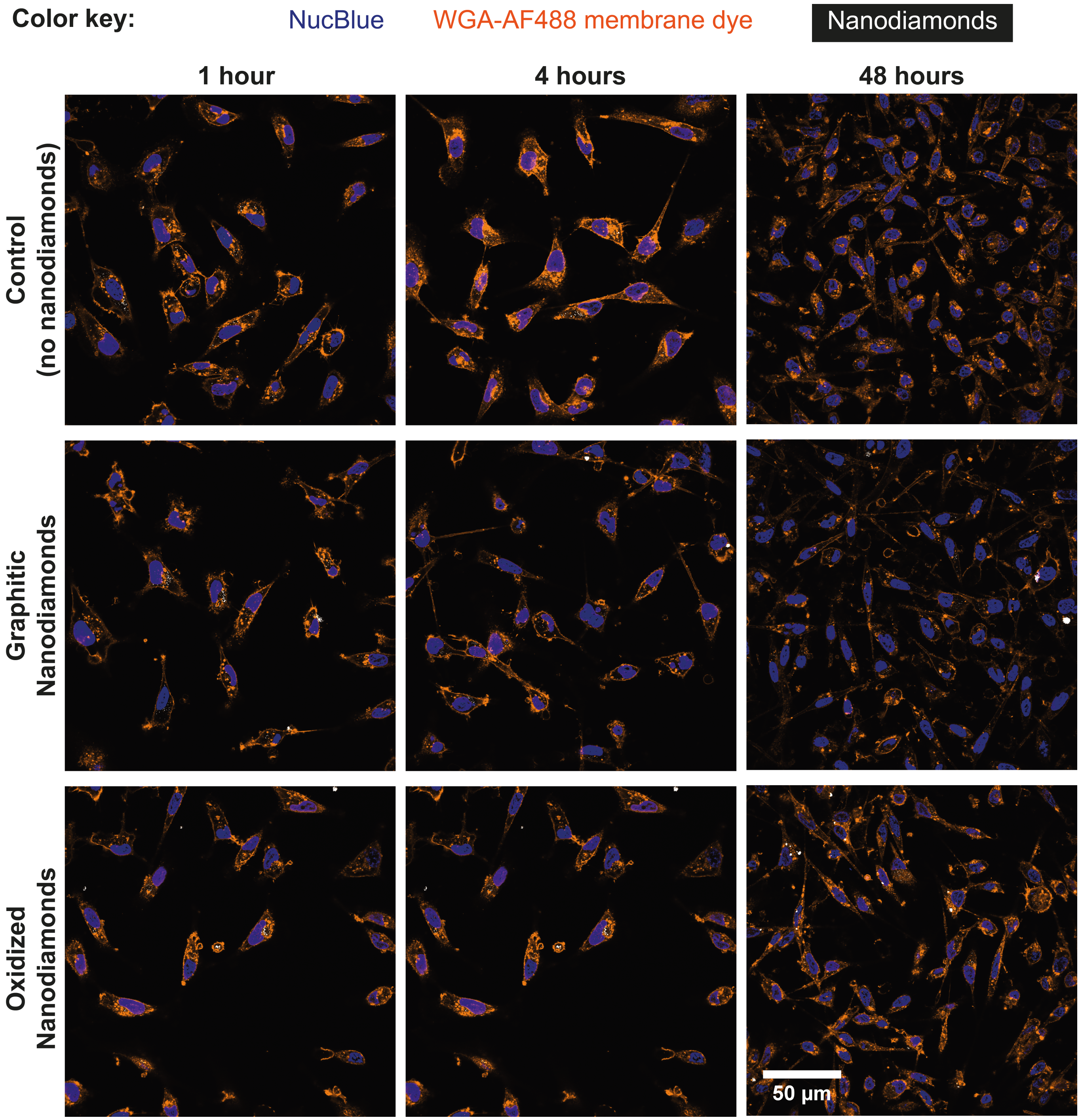

Figure S4: MDA-MB-231 confocal images of $1\,\mu g\,mL^{-1}$ nanodiamonds being internalized into cells. The cells were also stained to show membranes (WGA-AF488nm) and nuclei (NucBlue).

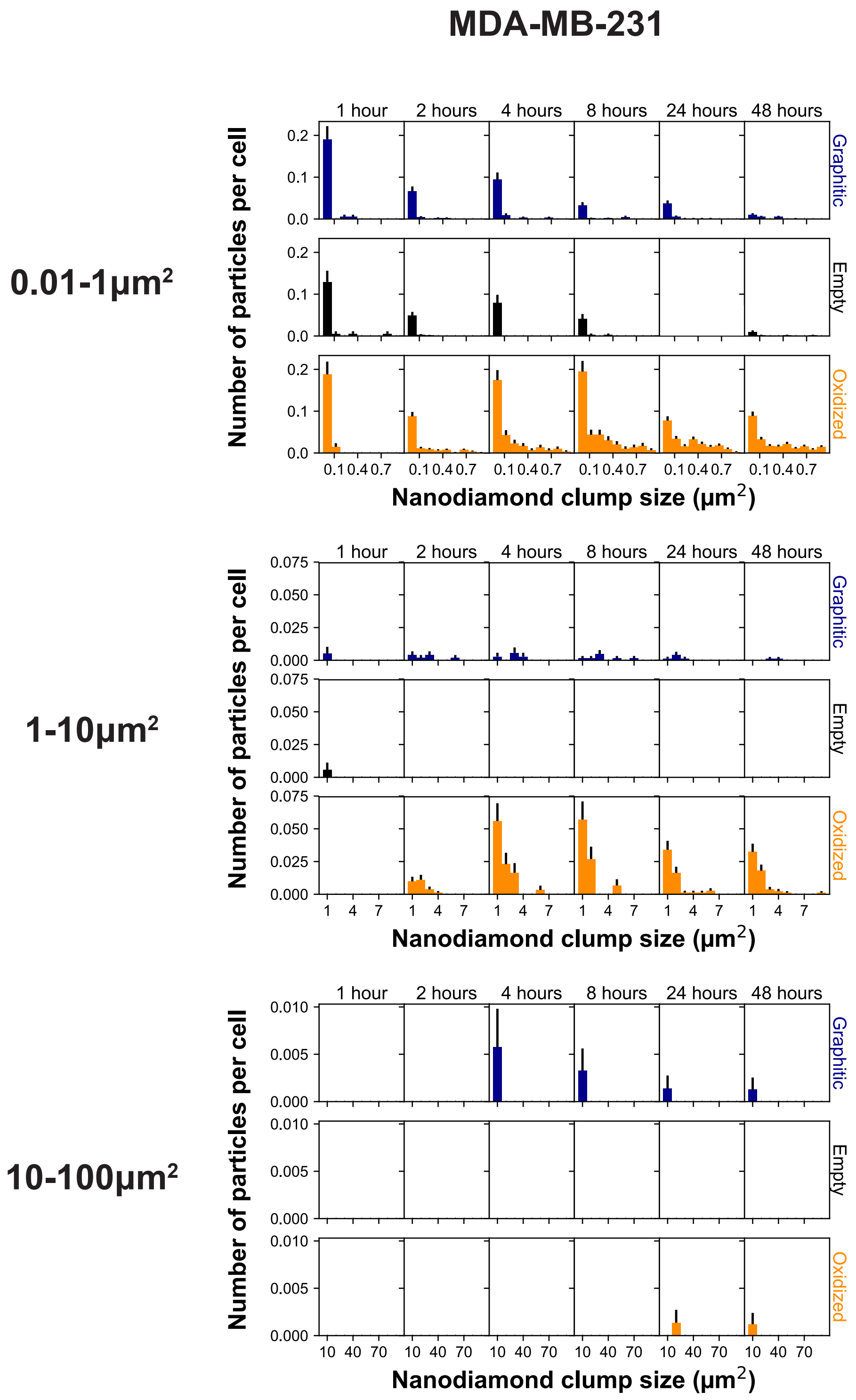

Figure S5: Size distribution histograms from the uptake experiment for the MDA-MB-231 cell line. The background for 24 h was observed to be anomalous and removed. Methods are described in main text.

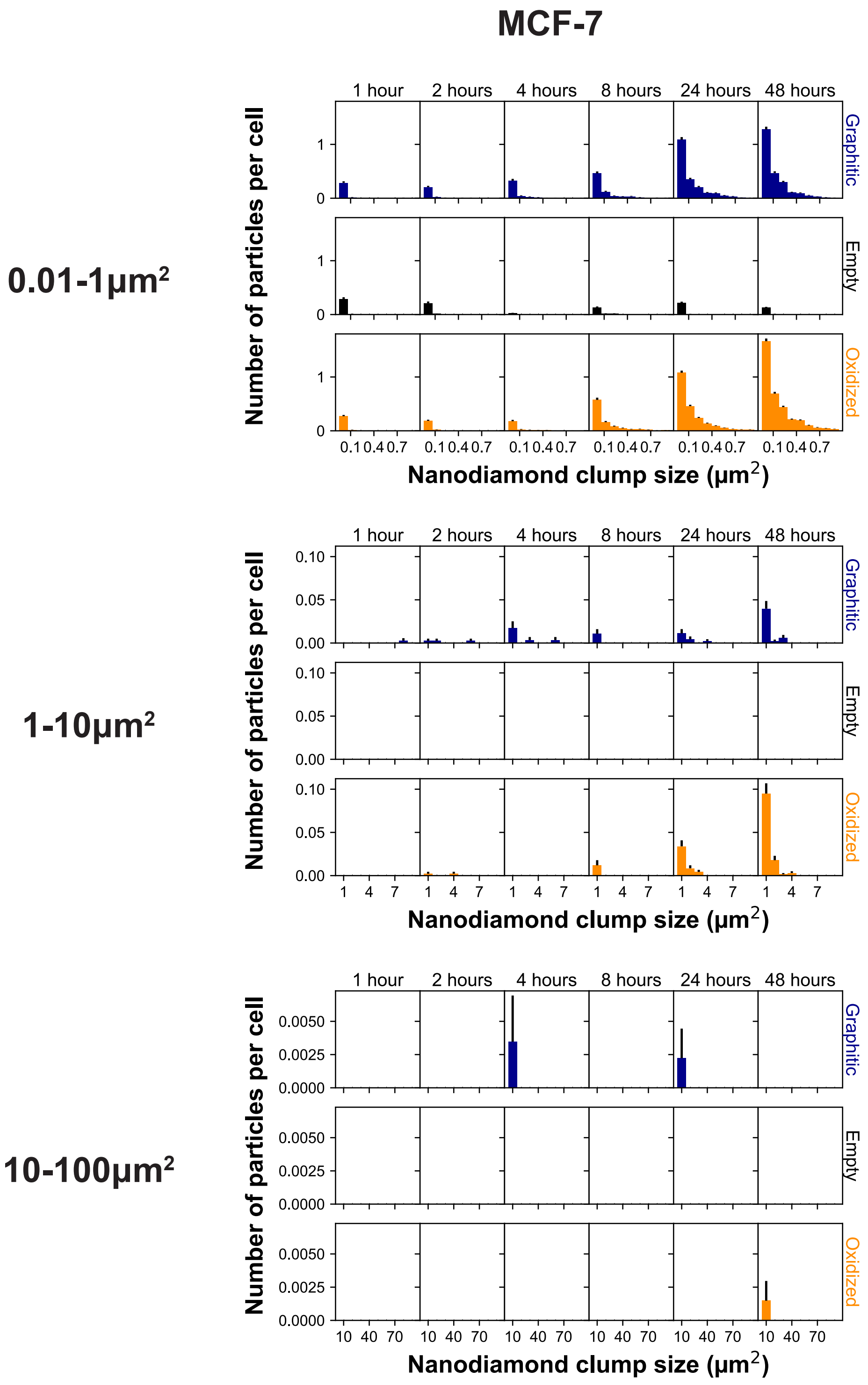

Figure S6: Size distribution histograms from the uptake experiment for the MCF-7 cell line. Methods are described in main text.
